# The parabrachial nucleus is a critical link in the transmission of short latency nociceptive information to midbrain dopaminergic neurons

**DOI:** 10.1016/j.neuroscience.2010.03.049

**Published:** 2010-06-16

**Authors:** V. Coizet, E.J. Dommett, E.M. Klop, P. Redgrave, P.G. Overton

**Affiliations:** a Institut National de la Santé et de la Recherche Médicale U836, Grenoble Institute of Neurosciences, Joseph Fourier University, Grenoble 38042, France; b Department of Life Sciences, The Open University, Milton Keynes, MK7 6AA, UK; c Department of Neuroscience, Anatomy Section, University Medical Center Groningen, University of Groningen, 9700 AD Groningen, the Netherlands; d Department of Psychology, University of Sheffield, Western Bank, Sheffield, S10 2TN, UK

**Keywords:** nociception, extracellular recording, tract tracing, rat, ANOVA, analysis of variance, BDA, biotinylated dextran amine, BSA, bovine serum albumin, DA, dopaminergic, DAB, diaminobenzidine, FLI, fos-like immunoreactivity, NHS, normal horse serum, PB, phosphate buffer, PBN, parabrachial nucleus, PBS, phosphate buffered saline, PHA-L, *Phaseolus vulgaris* leukoagglutinin, PPTg, pedunculoponting tegmental nucleus, PSTH, peri-stimulus time interval histogram, RMTg, rostromedial tegmental nucleus, SD, standard deviation, SNPc, substantia nigra pars compacta, SNPr, substantia nigra pars reticulata, TH, tyrosine hydroxylase, TX, Triton X 100, VTA, ventral tegmental area

## Abstract

Many dopaminergic neurons exhibit a short-latency response to noxious stimuli, the source of which is unknown. Here we report that the nociceptive-recipient parabrachial nucleus appears to be a critical link in the transmission of pain related information to dopaminergic neurons. Injections of retrograde tracer into the substantia nigra pars compacta of the rat labelled neurons in both the lateral and medial parts of the parabrachial nucleus, and intra-parabrachial injections of anterograde tracers revealed robust projections to the pars compacta and ventral tegmental area. Axonal boutons were seen in close association with tyrosine hydroxylase-positive (presumed dopaminergic) and negative elements in these regions. Simultaneous extracellular recordings were made from parabrachial and dopaminergic neurons in the anaesthetized rat, during the application of noxious footshock. Parabrachial neurons exhibited a short-latency, short duration excitation to footshock while dopaminergic neurons exhibited a short-latency inhibition. Response latencies of dopaminergic neurons were reliably longer than those of parabrachial neurons. Intra-parabrachial injections of the local anasethetic lidocaine or the GABA_A_ receptor antagonist muscimol reduced tonic parabrachial activity and the amplitude (and in the case of lidocaine, duration) of the phasic response to footshock. Suppression of parabrachial activity with lidocaine reduced the baseline firing rate of dopaminergic neurons, while both lidocaine and muscimol reduced the amplitude of the phasic inhibitory response to footshock, in the case of lidocaine sometimes abolishing it altogether. Considered together, these results suggest that the parabrachial nucleus is an important source of short-latency nociceptive input to the dopaminergic neurons.

Dopamine-mediated transmission has been implicated in a number of human clinical disorders as well as in a wide range of normal brain functions. Typically, dopaminergic (DA) neurons exhibit a highly stereotyped, short latency (<100 ms), short duration (∼100 ms) population response to unpredicted stimuli in a variety of modalities that are salient by virtue of their novelty, intensity or reward value (Freeman and Bunney, 1987; Horvitz et al., 1997; Schultz, 1998). Dopaminergic neurons also respond to noxious stimuli in a wide range of species, including the rat (e.g. Coizet et al., 2006), rabbit (Guarraci and Kapp, 1999) and monkey (e.g. Schultz and Romo, 1987). In the rat, noxious stimuli produce a short-latency increase, or more commonly decrease, in discharge frequency (Tsai et al., 1980; Maeda and Mogenson, 1982; Mantz et al., 1989; Gao et al., 1990; Ungless et al., 2004; Coizet et al., 2006).

While much is known about many aspects of the ascending dopamine systems, surprisingly little is known about the sensory inputs that phasically modulate their activity. We have recently shown that a subcortical visual structure, the midbrain superior colliculus (SC), is the primary, if not the exclusive, source of short-latency visual input to midbrain DA neurons (Comoli et al., 2003; Dommett et al., 2005), possibly mediated in part by the tectonigral projection—a direct projection from the SC to the substantia nigra pars compacta (SNPc) and ventral tegmental area (VTA), which innervates DA neurons in these regions (Comoli et al., 2003; McHaffie et al., 2006; May et al., 2009). However, although the SC contains neurons which respond to noxious stimuli (e.g. Stein and Dixon, 1979), in contrast to vision, it does not appear to transmit pain-related information to DA neurons (Coizet et al., 2006).

Although the source of the afferent inputs which relay pain-related information to DA neurons is still uncertain, during our retrograde anatomical work on the tectonigral projection, which involved the placement of tracer injections in the SNPc, we noticed numerous retrogradely labelled cells in the mesopontine parabrachial nucleus (PBN). The PBN is a major central target for ascending nociceptive information from the spinal cord (Hylden et al., 1989; Craig, 1995; Klop et al., 2005), which raises the possibility that the PBN may provide nociceptive signals to DA neurons. This was investigated initially by using tract tracing experiments to confirm the existence of a direct parabrachio–nigral projection and explore its properties. Following these, we used electrophysiological procedures to examine the effects of chemical inactivation of the PBN (using the local anaesthetic lidocaine or the GABA_A_ receptor antagonist muscimol) on the phasic responses of DA neurons to noxious stimuli.

## Experimental procedures

All aspects of these studies were performed with Home Office approval under section 5(4) of the Animals (Scientific Procedures) Act 1986, and experimental protocols received prior approval from the Institutional Ethics Committees.

### Anatomical experiments

#### Surgical preparation

For the retrograde and anterograde tract tracing experiments, 12 male Hooded Lister rats (398–672 g) were anaesthetized with an i.p. injection of a mixture of ketamine (Ketaset, 0.765 ml/kg) and xylazine (Rompun, 1.1 ml/kg) and mounted in a stereotaxic frame (David Kopf Instruments, Tuajanga, CA, USA) with the skull level. Body temperature was maintained at 37 °C with a thermostatically controlled heating blanket.

#### Retrograde tracer injections

In the first group of rats (*n*=4), the retrograde tracer fluorogold (Fluorochrome LLC, Denver, CO, USA) was injected into the SNPc (5.2–6.04 caudal to bregma, 1.4–2.6 mm lateral to midline, 7.3–8.2 mm below the brain surface) as a 4% solution in distilled water (45–100 nl) via a glass micropipette using a compressed air injection system. As described in a previous paper (Coizet et al., 2007), these injections were made under electrophysiological guidance to improve the successful placement of the tracer. Briefly, the glass pipette was joined to a Parylene-C coated tungsten electrode (2 MΩ; A-M Systems Inc., Carlsborg, WA, USA) and the assembly lowered into the ventral midbrain until the electrophysiological record showed an absence of activity (usually at a depth of around 8.0 mm), corresponding to the medial lemniscus. Shortly after, the record typically revealed the presence of fast firing activity characteristic of neurons in the substantia nigra pars reticulata. Tracer injections were made as soon as this fast activity was encountered.

#### Retrograde tracer histology and analysis

After allowing 7 days for the transport of tracers, animals were re-anaesthetized with pentobarbitone and perfused transcardially with warm saline (40 °C) followed by 4% paraformaldehyde in phosphate buffer (PB) (pH 7.4). The brains were placed in 10% formalin for 4 h before being cryoprotected by immersion in sucrose solution (20% in 0.1 M PB) overnight. The next day, coronal sections (30 μm) were cut on a freezing microtome and collected directly onto slides, allowed to dry in a light protected box and coverslipped in DPX.

The injection sites and retrogradely labelled cells in the PBN were examined with a fluorescent microscope equipped with episcopic illumination (Nikon Eclipse E800M, Kingston-upon-Thames, UK) and UV excitation filter (330–380 nm). The location of retrogradely-labelled neurons was plotted on three coronal sections through the PBN separated by ∼0.5 mm (equivalent to 8.8, 9.3 and 9.8 mm caudal to bregma, corresponding to anterior, central and posterior regions of the PBN respectively). A series of digital images (magnification×100) were taken using an RT Colour Spot camera (Diagnostic Instruments Inc., Sterling Heights, MI, USA) and imported into a graphics program (Macromedia Freehand, Adobe, San Jose, CA, USA) where they were montaged. Quantitative differences in cell counts within the PBN were assessed by repeated measures ANOVA (factors Laterality [levels: Lateral, Medial] and anterior–posterior position [levels: Anterior, Central, Posterior; accepted significance level *P*<0.05, 2 tailed).

#### Anterograde tracer injections

In a second group of rats, single injections of the anterograde tracers biotinylated dextran amine (BDA, Sigma-Aldrich; *n*=5) or *Phaseolus vulgaris* leucoagglutinin (PHA-L, Vector Laboratories, Peterborough, UK; *n*=3) were made into the PBN. An angled approach was used, with the injector tilted caudally by 35°, entering the brain at 11.2 mm caudal to bregma and 2.0 mm lateral to midline, after which it was inserted 6.0 mm below the brain surface. BDA (10% in phosphate buffer; PB) was pressure ejected in volumes of 30–90 nl via a glass micropipette (20 μm diameter tip) using a compressed air injection system, while PHA-L was ejected iontophoretically (5 μA anodal current applied to a 2.5% solution in PB, 7 s on/off for 15–20 min). After allowing 7 days for the transport of tracers, animals were re-anaesthetized with pentobarbitone and perfused transcardially with saline followed by 4% paraformaldehyde in PB (pH 7.4). The brains were placed immediately in 10% formalin for 4 h before being cryoprotected by immersion in sucrose solution (20% in 0.1 M PB) overnight. The next day, two series of coronal or sagittal sections (30 μm) were cut on a freezing microtome and collected in 0.1 M PB. Both series were processed to reveal the anterograde tracers, however the second series was subjected to an additional step, in which they were processed for tyrosine hydroxylase (TH).

#### Anterograde tracer histology and analysis

To reveal the tracers (BDA and PHA-L), free-floating sections were washed with 0.1 M PB followed by 0.1 M PB containing 0.3% Triton X-100 (PB-TX) for 30 min. For animals injected with PHA-L, the sections were incubated overnight in primary antibody solution (goat anti-PHA-L, 1:800–1,000 dilution, Vector Laboratories). The next day, sections were washed with PB-TX and incubated for 2 h in biotinylated rabbit anti-goat IgG (1:100, Vector Laboratories, in PB-TX containing 2% normal rabbit serum) for PHA-L. After 30 min of washing, all the sections from PHA-L and BDA animals were incubated in Elite Vectastain ABC reagent (Vector laboratories, 1:100 in PB-TX) for 2 h. The peroxidase associated with the tracers was revealed by reacting tissue with H_2_O_2_ for approximately 1 min using nickel-enhanced diaminobenzidine (DAB) as the chromogen for BDA (black reaction product), while PHA-L was revealed by incubation with VIP (Vector Laboratories) chromogen (purple reaction product). Finally, sections were washed in PB, and the first series were mounted on gelatin-coated slides, dehydrated in graded dilutions of alcohol, cleared in xylene and coverslipped using DPX.

The second series of sections were processed for TH as follows: Sections were incubated overnight with the primary mouse monoclonal antibody diluted 1:3000 (Roche Diagnostics, Lewes, UK) in 0.1 M PB-Trition-X 100 (TX) 0.3% with 1% bovine serum albumin (BSA) and 2% normal horse serum (NHS). The following day, sections were washed in 0.1 M PB and the secondary antibody, biotinylated antimouse made in horse (in a dilution of 1:1000 in 0.1 M PB-TX 0.3% with 2% NHS), was applied for 2 h. Following further washes in 0.1 M PB, the sections were exposed to the elite Vectastain ABC reagent (Vector Laboratories) diluted 1:100 in PB-TX 0.3% for 2 h. Again following washes in 0.1 M PB, immunoreactivity was revealed by exposure to VIP (Vector laboratories) for 3 min followed by several washes in 0.1 M PB to stop the reaction. Slices were then mounted onto gelled slides, dehydrated through alcohols and cleared in xylene before being coverslipped with DPX.

Following injections of anterograde tracers into the PBN, three coronal sections through the SNPc/VTA, approximately equivalent to 4.8, 5.3 and 5.8 caudal to bregma in the atlas of Paxinos and Watson (1998); corresponding to rostral SNPc, central SNPc/rostral VTA and central SNPc/caudal VTA respectively), or three sagittal sections approximately equivalent to 0.9, 1.9 and 2.9 mm lateral to midline (corresponding to VTA, medial SNPc and lateral SNPc respectively) were selected for analysis. Photomicrographs of injection sites in the PBN and of the SNPc/VTA on each section were taken using an RT Colour Spot camera (Diagnostic Instruments Inc.) and Nikon Eclipse E800M microscope (Nikon Instruments). Fibres and terminals associated with the injections were traced with the aid of a graphics program (Macromedia Freehand, Adobe).

### Electrophysiological experiments

#### Surgical preparation

Seventeen female Hooded Lister rats (220–300 g) were anaesthetized with an i.p. injection of urethane (ethyl carbamate, Sigma-Aldrich; 1.25 g/kg as a 25% aqueous solution) and mounted in a stereotaxic frame with the skull level. Body temperature was maintained at 37 °C with a thermostatically controlled heating blanket. Two stainless steel electrodes (E363-1, Plastics One, Roanoke, VA, USA) were inserted into the left hindpaw, one under the skin of the plantar surface of the foot and the other under the skin of the medial aspect of the lower leg/ankle. Craniotomies were then performed to allow access to the PBN and SNPc.

#### Recording and injection procedure

Extracellular single unit recordings were made from DA neurons located contralaterally to the stimulated hindpaw, using glass microelectrodes pulled via a vertical electrode puller (Narashige Laboratory Instruments Ltd. Tokyo, Japan) and broken back against a fire polished glass rod to a tip diameter of approximately 1 μm (impedances 5–20 MΩ, measured at 135 Hz in 0.9% NaCl). Electrodes were filled with 0.5 M saline and 2% Pontamine Sky Blue (BDH Chemicals Ltd., Poole, UK). After manufacture, the electrode was lowered to a position just dorsal to the SNPc (5.2–6.04 mm caudal to bregma, 1.5–2.6 mm lateral to midline, 7.2–8.0 mm ventral to the brain surface) with a hydraulic microdrive (Model 650, David Kopf Instruments).

Extracellular multiunit recordings were made from parabrachial neurons ipsilateral to the DA recording electrode using a tungsten electrode coupled to a 30 gauge stainless steel injector filled with either lidocaine (40 μg/μl in distilled water, Sigma-Aldrich, Poole, UK) or muscimol (0.25 μg/μl in saline, Sigma-Aldrich). Lateral separation between the electrode and the injector was 0.2–0.5 mm, with the electrode positioned 0.5 mm forward of the injector. Again, an angled approach was used, with the electrode tilted caudally by 35°, entering the brain at 11.0 mm caudal to bregma and 2.0 mm lateral to midline. Parabrachial neurons were encountered 5.6–6.2 mm below the brain surface. Intra-parabrachial microinjections were made (0.5 μl at a rate of 0.5 μl/min) via a 10 μl Hamilton syringe mounted on an infusion pump, connected to the injector by a length of plastic tubing.

Spike related potentials were amplified, band-pass filtered (300 Hz–10 kHz), digitized at 10 kHz and recorded directly onto computer disc using a Micro 1401 data acquisition system (Cambridge Electronic Design [CED] Systems, Cambridge, UK) running CED data capture software (Spike 2).

#### Stimulation procedure

Parabrachial neurons were identified by their response to noxious footshock induced by single pulses (0.5 Hz, 2 ms duration) at an intensity of 3.0–5.0 mA, around 3× the threshold intensity required for C-fibre threshold activation (Chang and Shyu, 2001; Matthews and Dickenson, 2001; Carpenter et al., 2003; Urch et al., 2003). Once the parabrachial electrode/injector had been positioned, the DA electrode was lowered until a putative DA neuron was identified on the basis of standard criteria (Grace and Bunney, 1983): long action potential duration (>2.0 ms), low firing rate (<10 Hz) and a firing pattern that consisted of irregular single spikes or bursts. Once encountered, the activity of the cell (and multiunit activity in the PBN) was recorded during the application of noxious footshock. Following a period of baseline response determination (120 trials), an injection of either lidocaine or muscimol was made into the PBN. Typically, a change in local parabrachial multiunit activity was seen within 60–120 s of the injection. Noxious electrical footshock stimulation was applied throughout this period, until either the effects of the drug wore off in the PBN, or the DA cell was lost. After a complete trial, further DA neurons were tested in the same way. Between 1 and 2 DA cells were tested in a single subject.

#### Histology

At the conclusion of an experiment, the final recording site for the DA recording electrode was marked by passing a constant cathodal current of 27.5 μA (constant current source: Fintronics Inc. Orange, CA, USA) through the electrode for a period of 30 min to eject Pontamine Sky Blue and the parabrachial recording site was marked by passing a 10 μA anodal current for 3 min through the tungsten recording electrode to create a small lesion. After marking the recording sites, animals were killed with an overdose of barbiturate and perfused transcardially with 400 ml of warmed saline (40 °C), followed by 400 ml of paraformaldehyde in phosphate buffer (PB, pH 7.4). Brains were removed and postfixed overnight in 4% paraformaldehyde at 4 °C, before being transferred into sucrose for 36 h. Serial coronal (50 μm) sections were cut on a vibratome. One series of sections were mounted on slides and processed with a Nissl stain (Cresyl Violet). A second series of sections were collected in 0.1 M PB and processed for TH using the method outlined earlier. Once sections had been processed, recording sites were reconstructed onto sections taken from the atlas of Paxinos and Watson (1998).

#### Data analysis

The waveform characteristics of recorded DA neurons were determined off-line from averaged records. Typically, 200–300 digitized spikes were averaged to produce a waveform average for each neuron. These averages were used to determine the width of the action potential according to the criteria of both Grace and Bunney (1983) and Ungless et al. (2004). For analysis of the firing pattern of DA neurons in response to the stimuli applied, spikes were separated from the background noise and stimulus artifact using template matching (Spike 2; CED). In the case of the parabrachial mulitunit data, data files were high pass filtered to attenuate the stimulus artifact and then thresholded using the WaveMark facility in Spike 2. Following this preprocessing, peristimulus time interval histograms (PSTHs) were constructed based on DA single unit and parabrachial multiunit data (binwidth 20 and 1 ms respectively). PSTHs were imported into an Excel program (Microsoft) (Peter Furness, Sheffield) which determined the following response characteristics before and after an injection of lidocaine/muscimol: (i) Baseline activity: the mean number of spikes per bin occurring during the 500 ms prior to the stimulation; (ii) Response latency: response onset was defined as the time point at which post-stimulation deviations in activity exceeded 1.96 standard deviations (SDs) of the pre-stimulation baseline (measured over 100–500 ms before the stimulus) for DA single units, and 3.00 SDs for parabrachial multiunit activity; there parameters (coupled with the binwidths above), which had to be tailored to the cell type, provided a principled measure of latency which closely matched estimates based on visual inspection; (iii) Response duration: response offset was defined as the time point at which post-stimulation activity returned to within the threshold values and response duration was the difference in time between response latency (onset) and offset; (iv) Response amplitude: the mean number of multi-unit spikes per bin between response onset and offset, minus the baseline mean for excitations and subtracted from the baseline mean for inhibitions.

When considering the effect of chemical modulation of the PBN on the responses of DA neurons to electrical stimulation, DA neuronal activity was analysed for the period over which the drug affected parabrachial activity. This period was defined as that when activity in the PBN (measured over the first 200 ms post-stimulation) deviated outside 1.96 SDs of baseline determined during the 60 stimulations predrug. Quantitative differences in response parameters were assessed with *t*-tests (accepted significance level *P*<0.05, 2 tailed).

## Results

### Retrograde anatomy

**Fig. 1:**
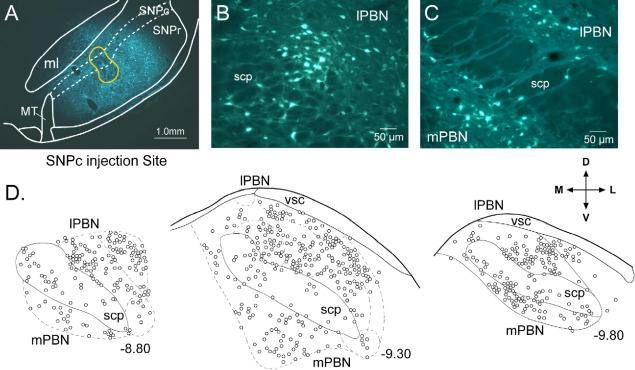
Parabrachial neurons retrogradely labelled from the substantia nigra pars compacta (SNPc) are found in both the lateral and medial parts of the parabrachial nucleus (PBN). (A) Photomicrograph of a fluorogold (retrograde tracer) injection site in the SNPc. (B) Photo-micrograph of retrogradely labelled neurons in the lateral parabrachial nucleus (lPBN) and (C) lateral and medial parabrachial nucleus (mPBN). (D) Quantitative plot of labelled neurons at different anterior-posterior levels of the PBN (numbers associated with each section indicate its location caudal to bregma in mm, according to the atlas of Paxinos and Watson, 1998). The directional arrows apply to (A–D); M, medial; L, lateral; D, dorsal; V, ventral. Additional abbreviations: ml, medial lemniscus; MT, medial terminal nucleus of the accessory optic tract; scp, superior cerebellar peduncle; SNPr, substantia nigra pars reticulata; vsc, ventral spino–cerebellar tract.

To confirm our previous informal observation that injections of retrograde tracer into the SNPc give rise to labelled cells in the PBN, small quantities of the retrograde tracer fluorogold were injected into the SNPc (Fig. 1A) and VTA. The general distribution of retrogradely labelled cells in the PBN was very similar following an injection in the lateral part of the SNPc, the central part of the SNPc, or VTA, and hence the projection appears to innervate the whole dopamine containing region of the ventral midbrain, but exhibits little topography. Retrogradely labelled neurons were found in all subnuclei of both the lateral and medial parts of the ipsilateral PBN and also within the fibres of the superior cerebellar peduncle (Fig. 1B, C). The Kolliker–Fuse nucleus was labelled weakly and inconsistently. Within the PBN itself, there was a tendency for the density of labelled cells to be greater laterally than medially (Fig. 1D), which coupled with the overall larger volume of the lateral PBN meant that significantly more retrogradely cells were found laterally than medially (two way repeated measures ANOVA, factor laterality; *F*=25.6, *df*=[1,3], *P*<0.05). This was especially true in the rostral and central parts of the nucleus (interaction between laterality and anterior–posterior position, *F*=10.1, *df*=[2,6], *P*<0.05; Fig. 1D).

### Anterograde anatomy

**Fig. 2:**
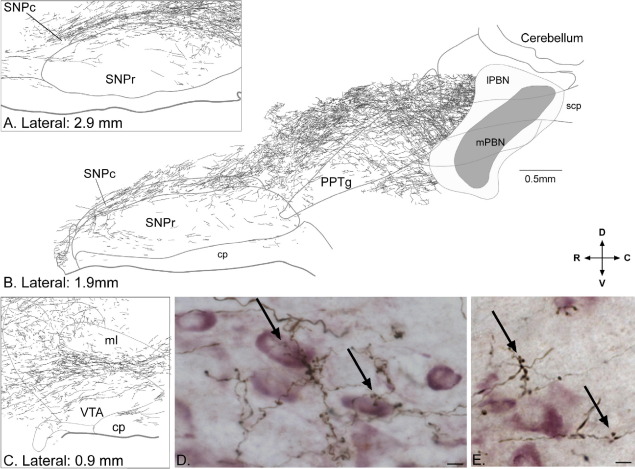
A robust projection from the parabrachial nucleus (PBN) to the substantia nigra pars compacta (SNPc) and ventral tegmental area (VTA) was evident. (A–C) Drawings based on photo-micrographs of parasagittal sections of rat brain following a large injection of the anterograde tracer *Phaseolus vulgaris* leucoagglutinin (PHA-L) in the PBN (shaded areas in the PBN in B). Dense labelling of fibres and terminal boutons can be seen in the SNPc (A, B) as well as in the VTA (C). (D, E). Examples of terminals and boutons anterogradely labelled from the PBN in relation to TH+ (presumed dopaminergic) neurons in the SNPc. Arrows indicate clusters of terminal boutons in close association with TH+ elements (D) and aggregating away from TH+ elements (E). Scale bar in (B) applies to (A–C) while scale bars in photomicrographs (D, E)=10 μm. In (A–C), laterality of the section is shown in mm according to the atlas of Paxinos and Watson (1998). The directional arrows apply to (A–E); R, rostral; C, caudal; D, dorsal; V, ventral. Additional abbreviations: cp, cerebral peduncle; lPBN, lateral parabrachial nucleus; ml, medial lemniscus; mPBN, medial parabrachial nucleus; PPTg, pedunculopontine tegmental nucleus; SNPr, substantia nigra pars reticulata; scp, superior cerebellar peduncle.

To add further support to the existence of a projection from the PBN to the SNPc and VTA, and to examine its relationship to DA neurons, we used anterograde tract-tracing techniques in combination with TH immunohistochemistry. Injections of the anterograde tracers PHA-L or BDA into the PBN revealed a robust direct projection to the ipsilateral SNPc and VTA (Fig. 2A–C). The pathway projects in an antero–dorsal direction from the PBN, passes through the caudal pedunculopontine tegmental nucleus (PPTg) and dorsal to the rostal PPTg, then curves ventrally to enter the caudal pole of the SNPc (Fig. 2A, B), and is thus best visualized in sagittal sections. Labelled fibres run the full rostro–caudal length of pars compacta (Fig. 2A, B), with fibres entering the medially located VTA at rostral levels (Fig. 2C). A few fibres continue forward into the hypothalamus. Comparatively sparse anterograde labelling was seen in the substantia nigra pars reticulata. Throughout the SNPc and VTA, numerous axonal boutons could be seen in close association with TH-positive perikarya and dendrites (Fig. 2D), as well as in regions devoid of TH immunostaining (Fig. 2E). Structures dorsally adjacent to the SNPc, including zona incerta, also contained anterogradely labelled boutons and axons. Both BDA and PHA-L produced qualitatively similar anterograde labelling.

### Electrophysiology

**Suppl. Fig. 1:**
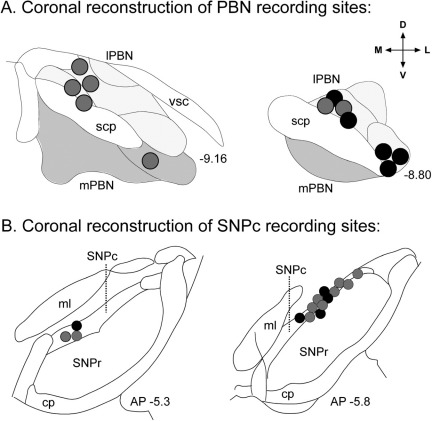


**Fig. 3:**
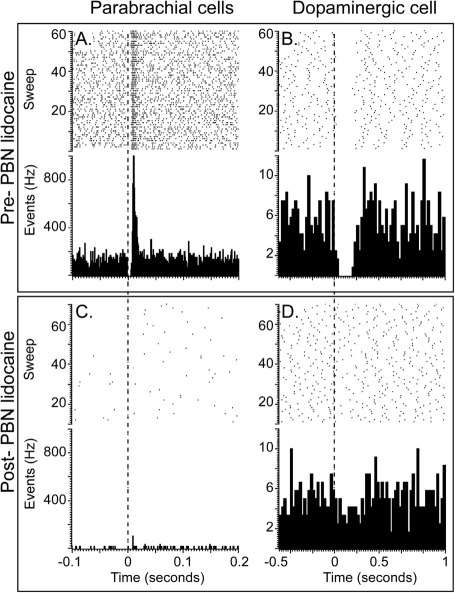
Effects of local intraparabrachial injections of lidiocaine on footshock-evoked multi-unit responses in the parabrachial nucleus (PBN) and in a single dopaminergic (DA) neuron. The graphs present raster displays and peri-stimulus histograms of single case data aligned on the presentation of 120 stimuli (0.5 Hz; vertical dotted line; stimulus artifacts have been removed for clarity—these did not overlap with responses in the PBN or in DA neurons). Prior to the injection of lidocaine, both the PBN and the DA neuron (A, B) were responsive to the footshock. Following the injection of lidocaine into the PBN, local neurons became unresponsive to the footshock (C) and so did the DA neuron (D).

Simultaneous recordings were made from the PBN and electrophysiologically identified DA neurons (*n*=15) in the SNPc of anaesthetized rats. Parabrachial sites were mainly located in the lateral part of the nucleus (Suppl. Fig. 1A) which receives the majority of the nociceptive input (Gauriau and Bernard, 2002) and provides the largest component of the parabrachio–nigral projection (see above). In all cases (*n*=12), noxious footshock produced a short latency (mean±1 SEM, 9.2±0.4 ms), short duration (15.5±2.1 ms) excitatory multi-unit response in the PBN (amplitude=268.6±32.8; Fig. 3A).

All the putative DA neurons sampled in the present study had firing rates (5.0±0.2 Hz) and action potential waveform durations (total duration=3.4±0.1 ms) which met the criteria of Grace and Bunney (1983) and virtually all (13/15; 86.7%) also met the waveform duration criterion of Ungless et al. (2004) (initial duration=1.2±0.0 ms). Furthermore, in all cases the neurons were located in the TH-immunoreactive region of the ventral midbrain corresponding to the SNPc (Suppl. Fig. 1B). The SNPc DA neurons sampled in the present study exhibited a phasic response to footshock (Fig. 3B), although in contrast to the PBN, in all cases this was a phasic suppression of activity. The latencies of the responses of DA neurons to electrical stimulation were significantly longer than those of the PBN (58.7±2.9 vs. 9.2±0.4 ms respectively; *t*[28]=17.1, *P*<0.001). The duration of the response in DA neurons was also significantly longer than that in the PBN (128.9±19.1 vs. 15.5±2.1 ms respectively; *t*[28]=5.9, *P*<0.001).

An injection of lidocaine adjacent to the parabrachial electrode decreased tonic activity in this structure, as measured by the decrease in baseline activity in the 500 ms prior to the application of each footshock (*t*[9]=4.0, *P*<0.01; Table 1). The application of lidocaine also suppressed the phasic nociceptive response in PBN neurons, in two cases abolishing it altogether (e.g. Fig. 3C). In the remaining cases, response amplitude and response duration were both significantly reduced (amplitude: *t*[7]=3.9, *P*<0.01; duration: *t*[7]=2.9, *P*<0.05). Intraparabrachial muscimol produced similar effects (Table 2)—reducing tonic activity (*t*[4]=3.0, *P*<0.05), and the amplitude of the phasic response to footshock (*t*[4]=5.0, *P*<0.05). The duration of the response however was not affected (*t*[4]=2.2, *P*>0.05). Neither lidocaine nor muscimol affected response latency (lidocaine: *t*[7]=0.2, *P*>0.05; muscimol: *t*[4]=0.2, *P*>0.05).

Following the depression of PBN neuronal activity by lidocaine, the firing rate of DA neurons was significantly reduced (*t*[9]=2.6, *P*<0.05; Table 1). In addition, there was a suppression of the phasic inhibitory response, which in two cases was completely abolished (e.g. Fig. 3D). In the remaining cells, response amplitude was significantly reduced (*t*[7]=8.2, *P*<0.001). Response duration and latency were unaffected (duration: *t*[7]=1.0, *P*>0.05; latency: *t*[7]=0.0, *P*>0.05). Muscimol produced a similar reduction in response amplitude (*t*[4]=4.5, *P*<0.05; Table 2), although this time duration was also reduced (*t*[4]=3.7, *P*<0.05). Again, these changes occurred without a change in response latency (*t*[4]=0.0, *P*>0.05), although in contrast to lidocaine, baseline firing rate was also unaffected by muscimol (*t*[4]=0.1, *P*>0.05). Considered together, these results suggest that the PBN is an important source of short-latency nociceptive input to the DA neurons in the SNPc.

## Discussion

The source of the afferent inputs which relay pain related information to DA neurons is uncertain. On the basis of previous informal anatomical observations, we explored the possibility that the PBN may provide such signals. Our results demonstrate that a direct projection exists between the PBN and the ventral midbrain and that inactivating the PBN attenuates and in some cases eliminates nociceptive responses in DA neurons. These findings will be explored more fully following the consideration of certain technical issues.

### Technical considerations

In the present study, responses to noxious footshock were recorded in both parabrachial and DA neurons. There are several reasons for considering the footshock we used to be frankly noxious: (i) The electrical stimulation parameters (2 ms, 3–5 mA) were based on previous work showing that stimulation at this intensity/duration produces Aδ and C-fibre induced responses in the anaesthetized rat spinal cord (Urch et al., 2003); (ii) Our previous work has shown that these stimulation parameters evoke fos-like immunoreactivity (FLI) in the medial part of ipsilateral layers I and II of the lumbar spinal cord (Coizet et al., 2006), the region specifically targeted by peripheral nociceptive afferents (Swett and Woolf, 1985; Besson and Chaouch, 1987). This pattern of evoked FLI is consistent with that reported for noxious stimulation by others—footpinch and noxious heat (Hunt et al., 1987; Bullitt, 1990); (iii) These stimulation parameters produce similar responses in putative DA neurons to those induced by a frankly noxious footpinch (Coizet et al., 2006). The above considerations suggest we are reporting important aspects of pain processing in both the PBN and ventral midbrain.

A second technical issue concerns the identification of putative DA neurons in our electrophysiological studies using electrophysiological criteria alone. Although the identity of the putative DA neurons in our studies cannot be confirmed with certainty, for the following reasons it is probably safe to assume that an overwhelming majority were DA. Firstly, all cells met the identification criteria of Grace and Bunney (Grace and Bunney, 1983), and the overwhelming majority met the more stringent criterion suggested by Ungless et al. (Ungless et al., 2004; although see Margolis et al., 2006). Secondly, all cells were located in the TH immunoreactive region of SNPc, corresponding to the A9 DA cell group (Lindvall and Björklund, 1974). Non-DA neurons in this region of the brain account for only a small proportion (∼20%; Matsuda et al., 1987) of the total.

### Dopaminergic and parabrachial responses to noxious stimulation

All DA neurons sampled in the present study responded to noxious footshock with an inhibition of firing. This predominance of inhibitory responses to noxious stimulation fits well with the findings of previous studies in the rat (Tsai et al., 1980; Maeda and Mogenson, 1982; Mantz et al., 1989; Gao et al., 1990; Ungless et al., 2004). The nociceptive response latencies of SNPc DA neurons to footshock (58.7±2.9 ms) were similar to those reported previously by us (63.5±3.2 ms, Coizet et al., 2006) and for responses to transcutaneous noxious electrical stimulation applied to “different parts of the body” (56.5±5.8; Gao et al., 1990). Importantly, the nociceptive latencies of DA neurons were substantially longer than those of parabrachial neurons to the same stimulus (9.2±0.4 ms), as would be the case if the PBN was responsible for providing DA neurons with afferent nociceptive information.

Intra-parabrachial lidocaine and muscimol injections produced parallel effects on parabrachial and DA neuronal responses to noxious stimulation. Both drugs decreased baseline parabrachial activity and the amplitude (and in the case of lidocaine, the duration) of the phasic response to noxious stimulation. Indeed, in some cases lidocaine abolished the phasic response altogether. Calculations based on Tehovnik and Sommer (1997) suggest that the lidocaine (and muscimol, which has similar properties in this regard, Martin, 1991) is likely to have spread a little less than 0.5 mm from the injection site, and hence the injections are likely to have been largely confined to the PBN. These manipulations of parabrachial activity led to a reduction in the amplitude of the inhibitory response to noxious stimulation in DA neurons. Again, with lidocaine, in some cases the phasic response was abolished altogether. Response duration was also reduced by muscimol, and baseline firing rate by lidocaine. Neither lidocaine nor muscimol affected the latency of parabrachial or DA responses. Our results suggest that the PBN influences both phasic and tonic activity in DA neurons. Those cases where suppression of the inhibitory response to noxious stimulation in DA neurons was incomplete following intra-parabrachial lidocaine probably reflect the incomplete suppression of the parabrachial response in those animals, possibly coupled with the injection failing to access parts of the PBN. The fact that lidocaine affected baseline firing rate in DA neurons whereas muscimol did not can potentially be explained by the differential impact of these drugs on baseline activity in the PBN. Lidocaine reduced parabrachial activity by 77.1% in contrast to the weaker 47.0% reduction caused by muscimol (see Tables 1 and 2). Given that changes in parabrachial activity were so closely mirrored by changes in DA activity, this provides convergent evidence (in conjunction with the latency differences discussed above) that pain-related information is transmitted to SNPc DA neurons from the PBN.

### Anatomy and function

Our anatomical experiments revealed the existence of a robust direct projection from the PBN to the SNPc and VTA. Saper and Loewy (1980) previously described such a projection in an early anterograde study, however the techniques they employed did not allow them to determine whether the label they observed following PBN injections arose from terminals or fibres of passage. Our results now confirm the existence of this pathway. Although the pathway emerges from both medial and lateral parts of the PBN, the lateral, nociceptive recipient (Gauriau and Bernard, 2002) part provides a larger component of the projection. Our light microscopic data suggest that the projection contacts both DA and non-DA elements in the SNPc and VTA. The majority of non-DA cells in both the SNPc and VTA are GABAergic (Nair-Roberts et al., 2008), and appear to regulate DA neurons (Gulley and Wood, 1971; Omelchenko and Sesack, 2009). In contrast, the majority of parabrachial neurons are glutamatergic (and not GABAergic; Guthmann et al., 1998; Yokota et al., 2007). Hence, excitatory parabrachial contacts onto GABAergic neurons in the SNPc and VTA provides a potential route by which pain can inhibit DA neurons (the predominant response in the rat, e.g. Coizet et al., 2006), while direct inputs to DA neurons may mediate the excitatory responses to noxious stimuli which occur in some DA neurons (e.g. Brischoux et al., 2009). As with the inhibitory response to noxious stimuli, preliminary evidence suggests that intra-parabrachial muscimol may also attenuate excitatory DA responses to noxious stimuli (Coizet et al., unpublished observations). Although our light microscopic data suggest that the projection from the PBN contacts both DA and non-DA elements in the SNPc and VTA, the fact that the predominant response in DA neurons to noxious stimulation is inhibitory in the present study and in earlier studies (see above) suggests that parabrachial inputs to the SNPc may preferentially target non-DA elements. The long latencies of the inhibitory responses in DA neurons to noxious stimulation vs the excitatory responses in parabrachial neurons (58.7 vs. 9.2 ms in the present study) may arise as a consequence of the fact that the inhibitory responses are generated indirectly, via a local circuit in the SNPc.

This direct projection to the vental midbrain may act in concert with the recently described nociceptive input to the VTA and SNPc from the rostromedial tegmental nucleus (RMTg; Jhou et al., 2009a,b). The RMTg does not itself receive a direct nociceptive input from the spinal cord (Gauriau and Bernard, 2002), but does receive an excitatory input from the PBN (Jhou et al., 2009a), and projects to DA neurons in both the SNPc and VTA (Jhou et al., 2009b). Since RMTg neurons projecting to the ventral midbrain—at least to the VTA—are primarily GABAergic (Jhou et al., 2009b), this may provide an additional route by which pain related information can inhibit DA neurons. Also, since RMTg neurons projecting to the VTA also terminate on non-DA (presumed largely GABAergic) neurons in the region (Jhou et al., 2009b), the inhibition of these cells following RMTg activation may produce some of the excitatory responses to noxious stimuli in VTA DA neurons (e.g. Brischoux et al., 2009). What functional need is subserved by having a direct and an indirect pain pathway from the PBN to the ventral midbrain is uncertain. However, the link in the RMTg provides a means by which the numerous brain regions which supply afferent inputs to the RMTg (see Jhou et al., 2009a) might modulate a component of the pain related signal going forward to DA neurons.

Since in some cases parabrachial inactivation abolished nociceptive responses in SNPc DA neurons altogether, the PBN may be the exclusive source of nociceptive input to these neurons, acting either directly or indirectly via a link in the RMTg. Although our data do not rule out the possibility that the nociceptive signal from the PBN is conveyed to DA neurons by a more indirect route, the most parsimonious interpretation of the current electrophysiological data is that these substantial, proximal routes provide the majority of the signal. One potential component of a more indirect route is the lateral habenula which contains nociceptive neurons (Benabid and Jeaugey, 1989) and has been hypothesized to provide nociceptive information to DA neurons (Brischoux et al., 2009). However, the fact that nociceptive responses in DA neurons survive its destruction (Gao et al., 1990) suggests that the habenula may simply modulate pain related responses arriving from elsewhere (as with the SC, Coizet et al., 2006). Indeed, existing evidence suggests that the lateral habenula may provide information to DA neurons concerning aversive, non-noxious stimuli, including the absence of an expected reward (Matsumoto and Hikosaka, 2007). If information about noxious stimuli is provided by the PBN (which does not receive an input from the lateral habenula, Tokita et al., 2009), this suggests that signals concerning these two types of negative outcome may be provided to DA neurons by different circuitry, an observation which is likely to be important for the debate about the neural separation of different classes of punishment signal (e.g. Boksem et al., 2008).

As well as receiving nociceptive information from the spinal cord, the PBN also receives inputs from widespread areas of the brain (Tokita et al., 2009), including the rostral part of nucleus of the solitary tract (Herbert et al., 1990), a brain area involved in the processing of gustatory information (Norgren and Leonard, 1973). Gustatory processing in the PBN is normally associated with an area which includes aspects of the lateral and medial subnuclei surrounding the superior cerebellar peduncle, as well as neurons within it (Karimnamazi and Travers, 1998), all of which according to our retrograde anatomical results project to the SNPc and VTA. Excitotoxic lesions of the PBN block the increase dopamine overflow in the forebrain produced by taste stimuli (Hajnal and Norgren, 2005), suggesting that the PBN transmits gustatory information to DA neurons. Although the transmission of gustatory information from the lateral PBN may be shared between the direct and indirect (via the RMTg) projections from the PBN to the ventral midbrain, the fact that projections from the PBN to the RMTg seem to arise largely from the lateral PBN (see Fig. 5 of Jhou et al. 2009a) suggests that there may also be a substantial unshared direct gustatory signal arising from the medial PBN.

In spite of much being known about the ascending dopamine systems, the function of the dopamine signal to the forbrain is still hotly debated. Recent data have led to the suggestion that DA neurons provide the brain's reinforcement learning mechanisms with a “reward prediction error” signal that may be used to adjust future behavioural response probabilities (Schultz, 2002). However, we have recently demonstrated that DA neurons are supplied with short-latency visual information by a relatively primitive subcortical neural system, the midbrain SC (Comoli et al., 2003; Dommett et al., 2005). This presents a major problem for the reward predication error hypothesis, because the restricted perceptual capacities of this system affords limited ability to discriminate rewarding from non-rewarding but otherwise neutral stimuli. The “reward status” of noxious stimuli is far less ambiguous than that of non noxious (rewarding/neutral) stimuli, so little processing is required to ascertain that status. However, the fact that a primitive, primary sensory structure provides pain related information to DA neurons provides convergent evidence that the short latency sensory information they receive is relatively unprocessed, which is inconsistent with the proposal that DA neurons exclusively signal aspects of reward.

## Figures and Tables

**Table 1:** Effects of intra-parabrachial lidocaine on the firing rate and parameters of the phasic response to noxious footshock in parabrachial and dopaminergic neurons

Lidocaine	Firing rate (Hz)	Latency (ms)	Amplitude	Duration (ms)
PBN				
Pre	173.6±35.6	9.8±0.6	253.3±58.9	15.5±3.4
Post	39.8±17.7⁎	9.6±0.4	51.2±23.4⁎	7.1±2.2⁎⁎
DA neurons				
Pre	5.3±0.2	53.8±4.1	5.0±0.2	172.5±21.6
Post	4.8±0.2⁎⁎	53.8±5.7	2.9±0.2⁎⁎⁎	149.0±25.7

Values shown are means±1 standard error of the mean of the various measures. Symbols indicate significant differences between pre and post drug measures: PBN, parabrachial nucleus; DA, dopaminergic; Pre, pre-drug; Post, post-drug.

⁎ *P*<0.01;

⁎⁎ *P*<0.05;

⁎⁎⁎ *P*<0.001.

**Table 2:** Effects of intra-parabrachial muscimol on the firing rate and parameters of the phasic response to noxious footshock in parabrachial and dopaminergic neurons

Muscimol	Firing rate (Hz)	Latency (ms)	Amplitude	Duration (ms)
PBN				
Pre	143.2±29.6	8.4±0.7	290.4±24.6	19.4±1.5
Post	76.1±13.2⁎	8.3±0.8	144.9±36.4⁎	13.3±1.9
DA neurons				
Pre	4.3±0.2	64.0±4.0	4.2±0.3	98.8±23.6
Post	4.3±0.3	64.0±6.9	2.5±0.5⁎	37.6±10.0⁎

Values shown are means±1 standard error of the mean of the various measures. Symbols indicate significant differences between pre and post drug measures: PBN, parabrachial nucleus; DA, dopaminergic; Pre, pre-drug; Post, post-drug.

⁎ *P*<0.05.
